# Integrated Multi-Terrain Locomotion Recognition and Knee-Exoskeleton Assistance for Load Carrying: A Feasibility Study

**DOI:** 10.3390/bios16100570

**Published:** 2026-10-10

**Authors:** Jun Liu, Yichen Lin, Yinuo Yao, Yu Zhu, Haoran Zhang, Xiangyang Wang, Jianquan Sun, Bo Yuan, Yue Ma

**Affiliations:** 1 National Earthquake Response Support Service, Beijing 100049, China; 2 College of Engineering, Southern University of Science and Technology, Shenzhen 518055, China; suat24060168@stu.suat-sz.edu.cn; 3 Guangdong Provincial Key Laboratory of Robotics and Intelligent Systems, Shenzhen Institute of Advanced Technology, Chinese Academy of Sciences, Shenzhen 518055, China; 4 Faculty of Computer Science and Artificial Intelligence, Shenzhen University of Advanced Technology, Shenzhen 518107, China; 5 Niudi Tech. Co., Ltd., No. 9 Jiubin Road, Jiulongpo District, Chongqing 400050, China

**Keywords:** knee exoskeleton, inertial measurement unit, locomotion-mode recognition, surface electromyography, load-carrying assistance

## Abstract

Multi-terrain load carriage changes knee motion and muscular demand, requiring assistance that adapts to the current locomotion task. This feasibility study evaluated an integrated pipeline in which five inertial measurement units (IMUs) and a quaternion-based dynamic temporal pooling Transformer (QDTPformer) selected task-specific dynamic movement primitive (DMP) references for powered knee-exoskeleton assistance. Six participants took part across the offline recognition and human-assistance evaluations. For level walking (Flat), ramp ascent (Ramp), and stair ascent (Stairs), QDTPformer achieved 95.74% offline test accuracy and 89.54%±1.07% mean window-level accuracy in leave-one-subject-out evaluation. Online recognition selected the corresponding DMP reference and assistance parameters. Phase-aligned DMP–knee correlations were 0.967, 0.944, and 0.777 for Flat, Ramp, and Stairs, respectively. The equally weighted mean surface electromyography (sEMG) root mean square (RMS) reduction across two muscles and three tasks was 8.42%, with task- and wearer-dependent responses. The main contribution is an end-to-end recognition-to-assistance pipeline evaluated across three terrains using both kinematic and muscle-activity outcomes.

## 1. Introduction

Lower-limb exoskeletons are wearable robotic systems that operate mechanically in parallel with the user’s lower limbs to transmit supportive forces or joint torques. They may be passive or powered and may target rehabilitation, mobility assistance, or physical augmentation [1,2,3]. Powered knee assistance is particularly relevant to multi-terrain load carrying because the knee contributes differently to body support, limb advancement, and elevation across level walking, ramp ascent, and stair ascent. Additional external mass further increases lower-limb mechanical demand and can alter joint moments, muscular recruitment, and gait strategy. These task-dependent changes in knee-flexion amplitude, foot clearance, stance-to-swing transition, and power timing make a single fixed assistance profile unlikely to remain appropriate across terrains. The present study therefore investigates a powered knee-assistance system that adapts its reference motion and assistance parameters to the recognized locomotion task.

Prior reviews established that effective lower-limb assistance depends on the coordinated design of the mechanical structure, sensing interface, and controller, rather than on any component in isolation [1,2,3]. Mooney et al. [4] further showed that autonomous portable assistance can reduce the metabolic cost of walking, demonstrating that wearable systems can provide measurable benefits beyond fixed laboratory devices.

Generating suitable assistance requires a reference motion and control parameters compatible with the user and current task. Wu et al. [5] showed that individualized gait-pattern generation can tailor reference trajectories to user-specific motion. Ma et al. [6] demonstrated that online gait planning can update movement references during weight transfer in paraplegic rehabilitation. Together, these studies motivate adapting knee trajectories and assistance timing to both terrain and wearer motion during load carrying.

The assistance profile must also account for task- and user-dependent responses. Human-in-the-loop studies showed that effectiveness is sensitive to the timing and magnitude of the applied profile [7,8]. Portable systems have extended assistance across walking and running, while real-world studies have highlighted the value of personalization [9,10]. Tortora et al. [11] further showed that lower-limb exoskeleton use can affect neural activity and gait classification. More recent context-aware approaches improved robustness under variable walking conditions and adapted assistance between level walking and stairs [12,13]. The take-home message from these studies is that adaptive assistance should be evaluated at the system level, including task recognition, joint motion, and user response. Joint kinematics quantify how the generated reference relates to actual motion, whereas surface electromyography (sEMG) provides complementary information about muscle-specific responses. Accordingly, the present evaluation combines locomotion recognition and assistance control with measurements of knee kinematics and surface electromyography.

Reliable locomotion sensing is the first stage of this process. Wearable systems may use mechanical, physiological, or environmental information, and each sensing modality has different practical characteristics. Novak and Riener [14] reviewed sensor-fusion approaches in wearable robotics and showed that multiple information sources can be combined to support movement-state estimation and control. Li et al. [15] developed a lower-limb exoskeleton human–machine interface based on surface electromyography and motor-imagery electroencephalography, illustrating the potential of physiological signals for high-level assistance commands. However, physiological sensing can be influenced by electrode placement, skin condition, signal quality, and subject-specific variability. Environment-facing sensors can provide terrain information, but their performance may depend on visibility, illumination, and scene structure. Inertial measurement units (IMUs), by contrast, are compact, inexpensive, easy to mount on body segments, and capable of continuous acquisition without requiring external infrastructure. Lara and Labrador [16] and Bulling et al. [17] reviewed wearable-sensor-based human activity recognition and established inertial sensing as a practical basis for continuous motion analysis.

IMU-based locomotion recognition is inherently a multichannel time-series classification problem. The sensor signals contain both the instantaneous configuration of body segments and their temporal evolution. Conventional statistical classifiers can describe relatively simple feature distributions, whereas deep temporal models can learn hierarchical representations directly from sequential inputs. Wang et al. [18] reviewed deep-learning methods for sensor-based activity recognition; Fawaz et al. [19] summarized deep-learning approaches for time-series classification, and Nweke et al. [20] discussed the principal algorithms and research challenges associated with mobile and wearable sensor networks. Ronao and Cho [21] applied deep neural networks to smartphone-sensor activity recognition, demonstrating the feasibility of learning discriminative representations from raw or minimally processed inertial sequences. Guan and Plötz [22] further investigated ensembles of deep long short-term memory networks for wearable activity recognition, highlighting the value of temporal modeling for variable human-motion patterns. These studies provide the methodological basis for using convolutional, recurrent, and attention-based networks to distinguish locomotion modes from multichannel wearable data.

For powered lower-limb devices, locomotion recognition must also operate continuously and provide decisions that can be used by the controller. Huang et al. [23] combined neuromuscular and mechanical information for continuous locomotion-mode identification in powered lower-limb prostheses. Young et al. [24] showed that incorporating time-history information improved intent recognition by representing the temporal progression of gait, and Varol et al. [25] demonstrated multiclass real-time intent recognition for a powered lower-limb prosthesis. More recent studies have focused on inertial sensing in exoskeleton-related multi-terrain tasks. Du et al. [26] investigated locomotion-mode recognition using inertial signals for a hip exoskeleton. Tang et al. [27] proposed a deep network combining dense feature extraction and recurrent temporal modeling for lower-limb-exoskeleton locomotion recognition. Ma et al. [28] addressed real-time continuous locomotion-mode recognition and transition prediction for humans wearing a lower-limb exoskeleton. These studies demonstrate that inertial time-series models can distinguish multiple locomotion modes and can be deployed in online wearable systems.

Nevertheless, high offline classification accuracy does not by itself establish an integrated assistance system. A recognition model normally produces a probability vector for every sliding window, and these window-level predictions may fluctuate because of sensor noise, transient movement, or similarity between adjacent locomotion patterns. Directly mapping each raw prediction to a controller state could cause unnecessary switching of reference trajectories or assistance parameters. The recognition output must therefore be converted into a temporally stabilized task decision before it is used by the assistance controller. Majority voting, confidence thresholds, hysteresis, and command interpolation can serve different roles in this process: voting suppresses isolated prediction changes, hysteresis prevents repeated switching near a decision boundary, and interpolation maintains continuity when the selected assistance mode changes. In this study, the decision-stabilization stage serves as the interface between window-level recognition and real-time assistance control.

The IMU representation also affects what information is available to the recognition model. A calibrated orientation quaternion describes the orientation of a body segment relative to its initialized posture and therefore retains the overall configuration associated with a locomotion task. However, absolute orientation alone may not fully represent the direction and rate of local movement within a gait cycle. Adjacent-frame relative rotation provides complementary information by describing short-term rotational changes between consecutive samples. Combining calibrated quaternions with adjacent-frame relative rotations allows the input to preserve both posture-related and motion-related information. For the three locomotion tasks—level walking (Flat), ramp ascent (Ramp), and stair ascent (Stairs)—this combination is useful because the tasks differ not only in mean segment orientation but also in the timing and coordination of pelvic, thigh, and shank movements. A temporal model must aggregate these changes over the observation window while avoiding the loss of brief but informative gait phases.

Transformer encoders offer a flexible mechanism for modeling long-range dependencies in multichannel sequences, but a classification model still requires an operation that converts the encoded sequence into a fixed-dimensional representation. Global average pooling summarizes the complete window and provides a stable description of the overall temporal pattern, but it assigns equal importance to every time point. In contrast, learnable temporal weighting can emphasize portions of the window that are more informative for distinguishing locomotion modes, such as periods of lower-limb elevation or support transition. Combining dynamically weighted temporal pooling with global average pooling can therefore retain both locally discriminative information and the overall gait context. In the present study, this principle is implemented in a quaternion-based dynamic temporal pooling Transformer (QDTPformer) using data from the Pelvis IMU and the bilateral thigh and shank IMUs.

After a stabilized locomotion mode has been obtained, the system must generate a task-appropriate motion reference and map it to assistance torque. Dynamic movement primitives (DMPs) represent complex movements using stable dynamical systems. Ijspeert et al. [29] established the DMP formulation for learning attractor models of motor behavior. Ude et al. [30] investigated task-specific generalization of discrete and periodic DMPs, and Matsubara et al. [31] developed parametric DMP learning from multiple demonstrations. These properties make DMPs suitable for representing periodic knee-joint trajectories while retaining explicit parameters for temporal scale, amplitude, and phase. In multi-terrain assistance, separate DMP references can be associated with level walking, ramp ascent, and stair ascent, while compliant spring–damper mapping converts the difference between the reference and actual knee motion into continuous assistance torque.

The present study connects subject-independent locomotion recognition to task-specific knee assistance through online mode selection, DMP reference generation, and torque control. Knee kinematics and muscle activity characterize the resulting operation.

We tested three feasibility hypotheses:

**Hypothesis 1 (H1).** 
*The five-IMU model can distinguish Flat, Ramp, and Stairs, including under leave-one-subject-out evaluation.*

**Hypothesis 2 (H2).** 
*Stabilized recognition output can drive task-specific DMP reference and assistance selection online.*

**Hypothesis 3 (H3).** 
*Paired 10 kg assistance conditions show task- and wearer-dependent sEMG root mean square (RMS) changes.*

The main contributions are as follows:

1. An integrated multi-terrain assistance framework was implemented by combining five-IMU wearable sensing, online locomotion-mode decision-making, Simulink-based control, and a quasi-direct-drive knee exoskeleton used as the experimental hardware platform.
2. A QDTPformer locomotion-recognition module was developed using calibrated orientation quaternions, adjacent-frame relative rotations, dynamic temporal pooling, and global temporal aggregation. The stabilized recognition output was mapped to task-specific DMP reference trajectories and assistance parameters, and subject-independent performance was additionally examined using leave-one-subject-out validation.
3. The integrated system was evaluated using offline recognition, online assistance operation, right-knee kinematics, DMP–knee trajectory correspondence, and descriptive surface electromyography of the right vastus lateralis and right biceps femoris.

## 2. Materials and Methods

### 2.1. Overall System Architecture

**Figure 1:**
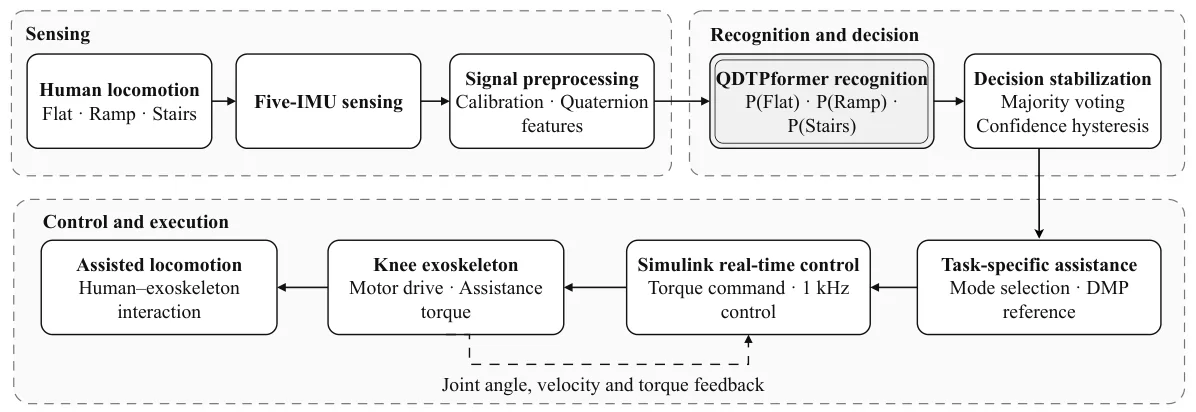
Overall architecture of the wearable sensing and locomotion-aware knee-exoskeleton assistance system. IMU, inertial measurement unit; QDTPformer, quaternion-based dynamic temporal pooling Transformer; DMP, dynamic movement primitive.

The system consisted of a quasi-direct-drive knee exoskeleton, a wearable sensing unit with five inertial measurement units (IMUs), a QDTPformer locomotion-mode recognition module, and a Simulink (R2022b; The MathWorks, Inc., Natick, MA, USA) real-time control platform (Figure 1). Motion data were acquired using the Pelvis IMU and the bilateral thigh and shank IMUs. After orientation calibration, feature construction, and sliding-window segmentation, the model outputs classification probabilities for level walking (Flat), ramp ascent (Ramp), and stair ascent (Stairs). Majority voting and confidence hysteresis converted the window-level predictions into a stabilized locomotion mode. The control platform then selected the corresponding DMP reference trajectory and assistance parameters and sent torque commands to the low-level controller.

### 2.2. Quasi-Direct-Drive Knee Exoskeleton System

#### 2.2.1. Mechanical Structure and sEMG Measurement Hardware

The quasi-direct-drive knee exoskeleton was developed by the authors’ team at the Shenzhen Institutes of Advanced Technology (SIAT) and served as the experimental hardware platform in this study. The exoskeleton consisted of a back-mounted power and control assembly, bilateral knee-joint actuation modules, adjustable thigh and shank linkages, and attachment structures. The back-mounted assembly integrated the battery, main controller, and power-management module. The linkage lengths were adjustable, and the device was secured to the thighs and shanks using straps.

**Figure 2:**
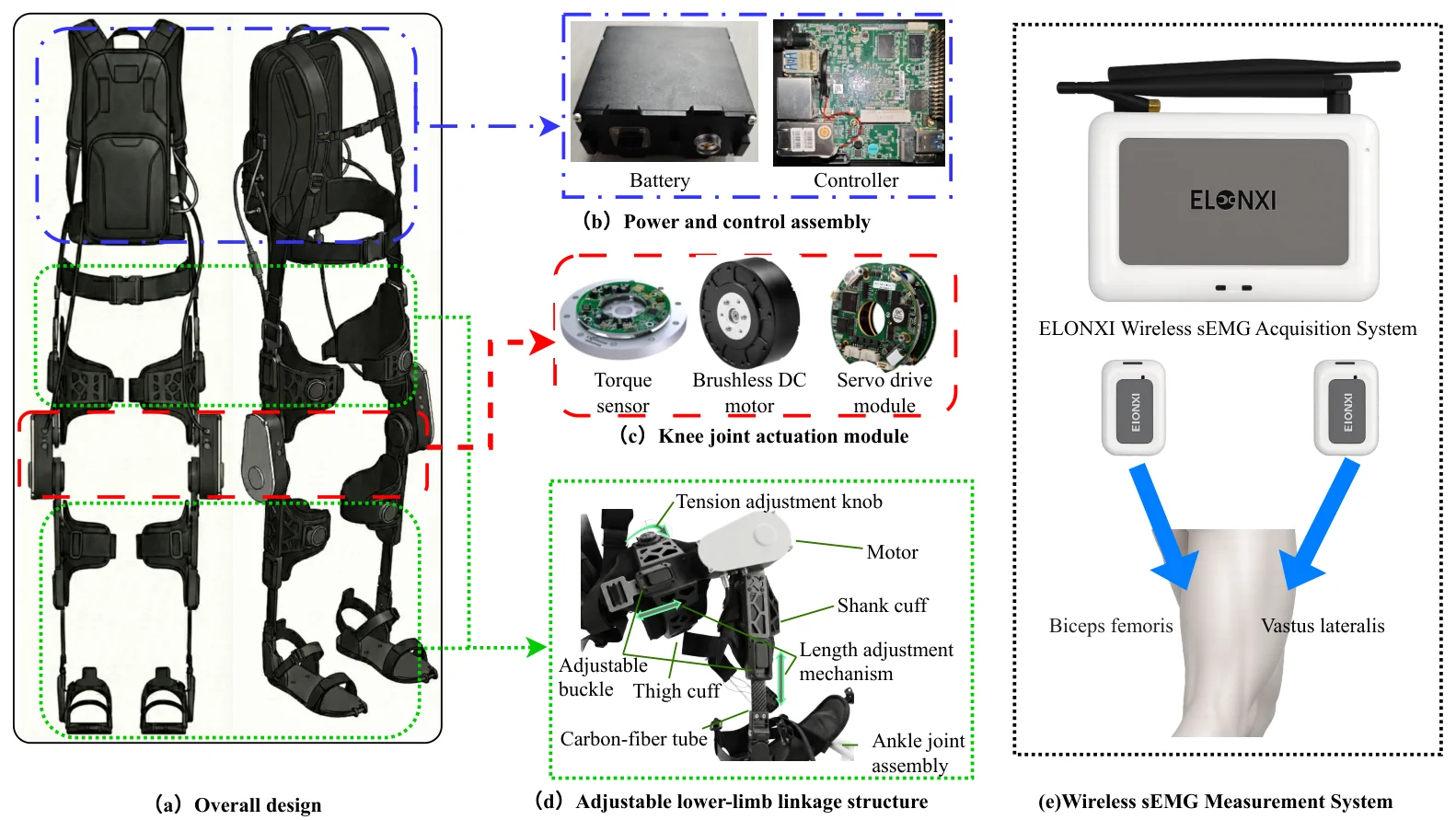
Quasi-direct-drive knee exoskeleton and wireless surface electromyography (sEMG) measurement system. (**a**) Overall design; (**b**) power and control assembly; (**c**) knee-joint actuation module; (**d**) adjustable linkage; (**e**) sEMG hardware and muscle locations. The blue, red, and green dashed boxes and arrows in (**a**) indicate the components detailed in (**b**), (**c**), and (**d**), respectively. The thin green leader lines in (**d**) identify the labeled mechanical components. DC, direct current.

Each knee-joint actuation module integrated a brushless motor, a low-ratio transmission, an encoder, a servo drive, and a torque sensor. A passive degree of freedom was provided at the ankle. The exoskeleton structure and principal specifications are shown in Figure 2 and Table 1, respectively.

Figure 2e shows the ELONXI wireless sEMG acquisition system (Hangzhou Jiaopu Technology Co., Ltd., Hangzhou, China) and the measurement locations over the right biceps femoris (BF) and right vastus lateralis (VL). This hardware was used only in the separate 10 kg load-carrying sEMG protocol described in Section 3.2.3.

#### 2.2.2. Electronics and Real-Time Control System

The back-mounted main controller received the locomotion mode, generated assistance commands, and managed the system state. The joint actuation units measured angle, angular velocity, and torque and executed current and torque control. The two levels communicated through EtherCAT, with a 1 kHz low-level control loop, as in a previously reported hierarchical wearable-robot controller [32]. The Simulink module performed locomotion recognition and assistance-parameter selection, while the joint drives executed the reference torque. LabVIEW was not used in the reported online implementation. Joint angle and torque feedback were used for low-level control, not as classifier inputs.

### 2.3. Five-IMU Wearable Sensing System

#### 2.3.1. Sensor Configuration and Placement

**Figure 3:**
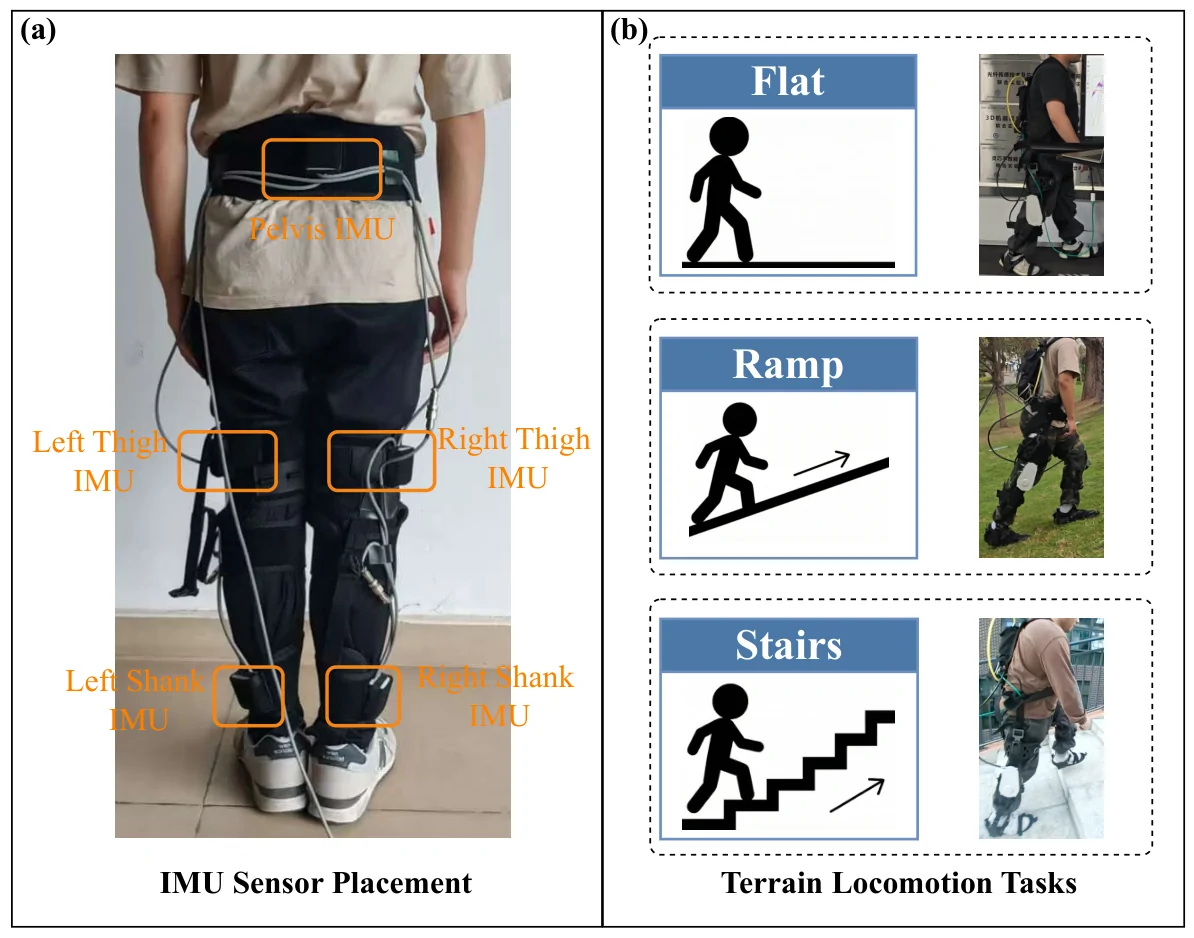
Wearable sensing system using five inertial measurement units (IMUs) and experimental tasks. (**a**) IMU placement; (**b**) Flat, Ramp, and Stairs.

The system used five WitMotion JY901S IMUs (WitMotion Shenzhen Co., Ltd., Shenzhen, China) with a sampling frequency of 100 Hz. The 100 Hz rate provides 10 ms inertial samples while remaining compatible with online wearable processing; comparable 100 Hz acquisition has been used in lower-limb-exoskeleton locomotion-recognition studies [27]. The Pelvis IMU was attached over the posterior pelvis; the remaining sensors were attached to the anterolateral regions of both thighs and the anterior or lateral regions of both shanks. Their positions are shown in Figure 3 and Table 2.

Each IMU provided tri-axial acceleration, tri-axial angular velocity, and an orientation quaternion. The classification input in this study used the orientation quaternion and its adjacent-frame relative-rotation features. The Pelvis IMU represented pelvic posture and the overall motion trend, whereas the bilateral thigh and shank IMUs described the swing and coordination of the lower-limb segments. The placement landmarks were selected to represent the pelvis and the major proximal/distal lower-limb segments surrounding the knee while preserving bilateral coordination information: the posterior-pelvis sensor captures global pelvic orientation, the thigh sensors capture femoral-segment swing, and the shank sensors capture distal segment motion relevant to knee flexion/extension patterns. This configuration covered the principal lower-limb segments associated with knee motion while retaining bilateral movement information.

#### 2.3.2. Orientation Initialization and Data Synchronization

Before each experiment, the subject stood naturally upright while the initial orientation of each IMU was recorded. The initialization was a brief static upright step used to acquire the reference orientation $q_{i}^{\mathrm{raw}}\left(0\right)$; it did not require a separate optimization or subject-specific training procedure. The raw orientation quaternion of the *i*th IMU at time *t* was expressed as

(1) $$q_{i}^{\mathrm{raw}}\left(t\right)={\left[\begin{array}{cccc} q_{w,i}^{\mathrm{raw}}\left(t\right) & q_{x,i}^{\mathrm{raw}}\left(t\right) & q_{y,i}^{\mathrm{raw}}\left(t\right) & q_{z,i}^{\mathrm{raw}}\left(t\right) \end{array}\right]}^{T},\;i=1,\ldots ,5.$$

The initial orientation was used as the reference for relative-orientation calibration:

(2) $$q_{i}^{r}\left(t\right)={\left[q_{i}^{\mathrm{raw}}\left(0\right)\right]}^{-1}⊗q_{i}^{\mathrm{raw}}\left(t\right),$$

where ⊗ denotes quaternion multiplication. The calibrated quaternion was then normalized:

(3) $$q_{i}\left(t\right)=\frac{q_{i}^{r}\left(t\right)}{{∥q_{i}^{r}\left(t\right)∥}_{2}}.$$

Because the unit quaternions q and −q represent the same rotation, sign discontinuities may occur during continuous acquisition. Sign consistency was therefore enforced between adjacent samples:

(4) $$\mathrm{if}\;q_{i}^{T}\left(t\right)q_{i}\left(t-1\right)<0,\;q_{i}\left(t\right)\leftarrow -q_{i}\left(t\right).$$

For notational simplicity, the quaternion after initial-orientation calibration, normalization, and sign-continuity processing is denoted by $q_{i}\left(t\right)$ hereafter. No additional drift-compensation algorithm was applied in the subsequent processing pipeline beyond use of the orientation quaternion supplied by the IMU, the initial relative-orientation calibration, normalization, and sign-continuity correction described above. Data from the five IMUs were synchronized using a common timestamp and concatenated in a fixed order to form each frame. The sensor IDs, channel order, and coordinate definitions were kept identical in all experiments to ensure that the data structures used for offline training and online recognition were consistent.

### 2.4. Data Segmentation and Locomotion-Mode Recognition

#### 2.4.1. Sliding Windows and Feature Construction

The continuously acquired five-IMU data were segmented using a sliding window. Each window contained 200 consecutive samples, and the step between adjacent windows was 20 samples. Thus, after the initial window was filled, the locomotion mode was updated whenever 20 new samples were received. The *k*th input window was represented as

(5) $$X_{k}=\left[x_{k,1},x_{k,2},\ldots ,x_{k,200}\right]\in R^{35\times 200},$$

where $x_{k,t}$ denotes the feature vector formed from the five IMUs at sampling instant *t*.

The 35-dimensional feature vector at each sampling instant consisted of calibrated orientation quaternions and adjacent-frame relative rotations. For the *i*th IMU, the processed orientation quaternion $q_{i}\left(t\right)$ provided four channels. The five IMUs therefore contributed 20 orientation features that preserved the current posture of each body segment relative to the initialized posture.

To describe local orientation changes between adjacent sampling instants, the relative-rotation quaternion of each IMU was calculated as

(6) $$\Delta q_{i}\left(t\right)=\mathrm{conj}\left(q_{i}\left(t-1\right)\right)⊗q_{i}\left(t\right).$$

The vector part of $\Delta q_{i}\left(t\right)$ was multiplied by 2 to obtain three relative-rotation features:

(7) $$\Delta r_{i}\left(t\right)=2{\left[\begin{array}{ccc} \Delta q_{x,i}\left(t\right) & \Delta q_{y,i}\left(t\right) & \Delta q_{z,i}\left(t\right) \end{array}\right]}^{T}.$$

Under the small-angle assumption, $\Delta r_{i}\left(t\right)$ approximately represents the rotation-vector increment between adjacent frames. It corresponds to angular velocity only after further division by the sampling period. The relative-rotation features from the five IMUs contributed 15 channels. For the first frame of each sequence, no preceding frame was available; therefore, $\Delta r_{i}\left(1\right)=0$. Consequently, each frame was represented by a 35-dimensional feature vector, as summarized in Table 3.

The calibrated orientation quaternion represented the current orientation of a body segment relative to its initialized posture, whereas the adjacent-frame relative rotation represented local orientation change. The former retained the overall configuration of the body segments within the window, and the latter emphasized dynamic changes between adjacent sampling instants. Together, they provided complementary descriptions of posture and motion. Each feature channel was standardized using the mean and standard deviation of the training set:

(8) $${\tilde{X}}_{k,c,t}=\frac{X_{k,c,t}-\mu_{c}}{\sigma_{c}+\varepsilon },$$

where $\mu_{c}$ and $\sigma_{c}$ denote the mean and standard deviation, respectively, of channel *c* in the training set. The validation set, test set, and online experiments all used the statistics calculated from the training set.

#### 2.4.2. Locomotion-Mode Recognition Network

QDTPformer was used to classify level walking (Flat), ramp ascent (Ramp), and stair ascent (Stairs). It mapped the 35-dimensional input composed of calibrated orientation quaternions and adjacent-frame relative rotations to a 64-dimensional temporal feature space. Learnable positional embeddings were then added before the features were passed through two Transformer encoder layers.

**Figure 4:**
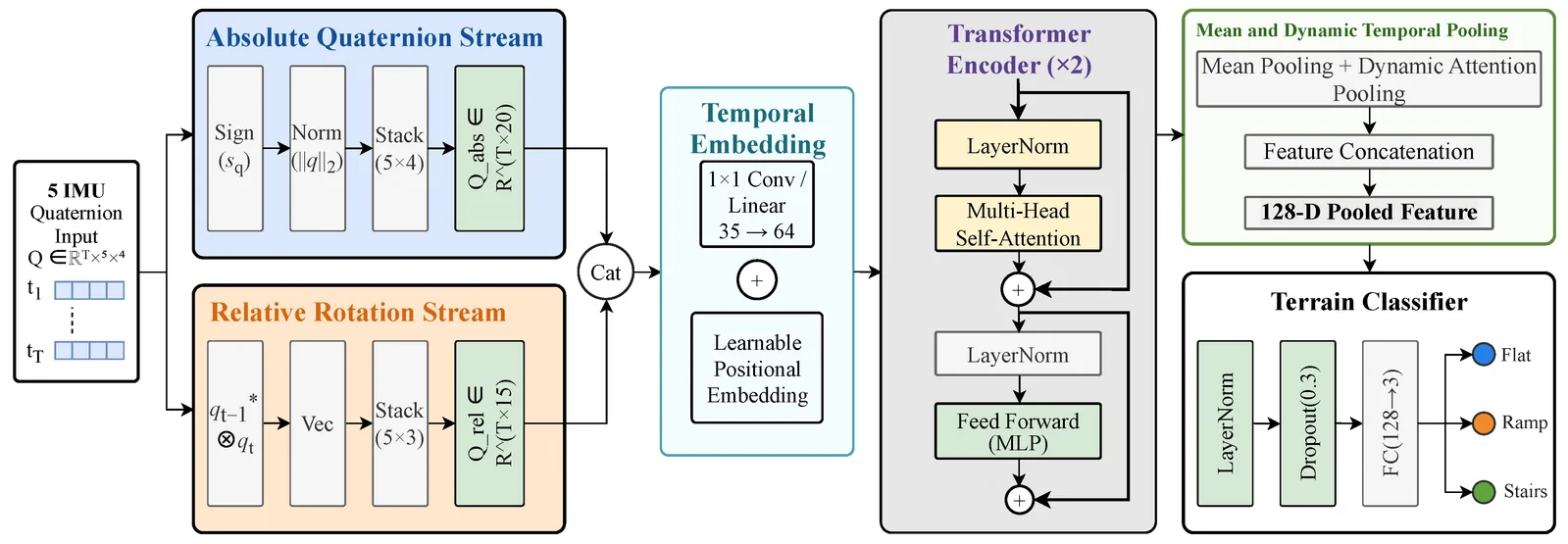
Overall architecture of the QDTPformer locomotion-mode recognition network. The superscript ∗ denotes quaternion conjugation. Conv, convolution; MLP, multilayer perceptron; FC, fully connected; and LayerNorm, layer normalization.

The encoded features were processed separately by learnable dynamic temporal pooling and global average pooling, and the resulting features were concatenated before classification. Here, “dynamic” indicates that the temporal weights were generated adaptively from the encoded features of the current input window rather than being fixed by time position or predefined coefficients. The network architecture and configuration are shown in Figure 4 and Table 4.

Let the Transformer encoder output be $H={\left[h_{1},\ldots ,h_{T}\right]}^{T}\in R^{T\times d}$. QDTPformer used a two-layer scoring network to calculate a scalar score for each time point and normalized the scores along the temporal dimension:

(9) $$s_{t}=v^{T}\tanh\left(Wh_{t}+b\right),\;\alpha_{t}=\frac{\exp(s_{t})}{\sum_{j=1}^{T}\exp\left(s_{j}\right)}.$$

The dynamic temporal pooling feature and global average feature were defined as

(10) $$z_{\mathrm{dyn}}=\sum_{t=1}^{T}\alpha_{t}h_{t},\;z_{\mathrm{avg}}=\frac{1}{T}\sum_{t=1}^{T}h_{t}.$$

Finally, $\left[z_{\mathrm{dyn}};z_{\mathrm{avg}}\right]$ was concatenated and passed through layer normalization, dropout, and a linear classification layer to produce the probabilities of the three classes.

### 2.5. Simulink-Based Online Recognition and Assistance-Mode Switching

After training, the best classification model and the normalization parameters obtained from the training set were deployed on the Simulink online control platform. The five-IMU data entered a sliding-buffer module. The first recognition result was produced after 200 samples had filled the buffer, and network inference was subsequently performed whenever 20 new samples were received. At 100 Hz, the window length was 2.0 s, and the subsequent update interval was 0.20 s. The model outputs the probability vector of the three locomotion modes:

(11) $$p_{k}={\left[\begin{array}{ccc} p_{k}^{\mathrm{Flat}} & p_{k}^{\mathrm{Ramp}} & p_{k}^{\mathrm{Stairs}} \end{array}\right]}^{T},\;{\hat{c}}_{k}^{\mathrm{raw}}=\arg\max_{c}p_{k}^{c}.$$

The system stored predictions from the three most recent windows. A class receiving at least two votes was selected as the candidate mode, and its candidate confidence was calculated as the mean probability of that class over the same three windows. The initialization threshold was 0.55. For switching to another mode, the threshold was 0.65, and the candidate mode had to be maintained for two consecutive decision cycles. This voting and confidence rule produced the stabilized mode used to select the Flat, Ramp, or Stairs DMP trajectory and assistance parameters.

During mode switching, the old and new assistance commands were interpolated as

(12) $$\tau_{\mathrm{ref}}\left(t\right)=\left[1-\beta (t)\right]\tau_{\mathrm{old}}\left(t\right)+\beta \left(t\right)\tau_{\mathrm{new}}\left(t\right),$$

where β(t) increased from 0 to 1 over a prescribed transition interval. The interpolated reference torque was then sent to the low-level torque controller.

### 2.6. DMP Reference-Trajectory Generation, Phase Adjustment, and Assistance Mapping

For each stabilized locomotion mode, the DMP represented the joint reference trajectory as a second-order spring–damper system modulated by a nonlinear forcing term:

(13) $$T_{\mathrm{act}}^{2}\ddot{y}=\alpha_{z}\left[\beta_{z}\left(g-y\right)-T_{\mathrm{act}}\dot{y}\right]+S_{\mathrm{amp}}f\left(\phi +\Delta \phi \right),\;T_{\mathrm{act}}=S_{\mathrm{time}}T_{0},$$

where *y* denotes the generated joint angle, *g* is the convergence target, and $\alpha_{z}$ and $\beta_{z}$ are system gains. $T_{0}$ and $T_{\mathrm{act}}$ denote the nominal and adjusted temporal scales, respectively. $S_{\mathrm{time}}$ and $S_{\mathrm{amp}}$ control the temporal scale and amplitude of the trajectory, respectively, and Δϕ denotes the phase-adjustment term. The nonlinear forcing term was obtained from a weighted set of Gaussian basis functions:

(14) $$\psi_{i}\left(x\right)=\exp\left[-h_{i}{\left(x-c_{i}\right)}^{2}\right],\;f\left(x\right)=x\frac{\sum_{i=1}^{M}\psi_{i}\left(x\right)w_{i}}{\sum_{i=1}^{M}\psi_{i}\left(x\right)},$$

where x=x(ϕ) is the canonical state variable driven by gait phase, and $c_{i}$, $h_{i}$, and $w_{i}$ denote the center, width, and learned weight of the *i*th basis function, respectively. The amplitude-normalized reference gait was used to update $w_{i}$, whereas $S_{\mathrm{time}}$, $S_{\mathrm{amp}}$, and Δϕ adjusted the temporal scale, amplitude, and phase, respectively. The phase-adjustment term, phase events, DMP trajectory, and control variables were recorded synchronously at a sampling interval of 0.001 s.

The reference angle and angular velocity output by the DMP were denoted by $\left(\theta_{\mathrm{ref}},{\dot{\theta }}_{\mathrm{ref}}\right)$. The low-level controller mapped the kinematic error to assistance torque through a virtual spring–damper relation:

(15) $$\tau_{\mathrm{cmd}}=K_{p}\left(\theta_{\mathrm{ref}}-\theta_{\mathrm{act}}\right)+K_{d}\left({\dot{\theta }}_{\mathrm{ref}}-{\dot{\theta }}_{\mathrm{act}}\right),$$

where $K_{p}$ and $K_{d}$ denote the equivalent stiffness and damping coefficients, respectively. Through this mapping, the DMP provided a task-specific kinematic reference, while the virtual spring–damper controller generated continuous assistance torque from the difference between the reference and actual knee motion, thereby connecting the trajectory-generation layer to the low-level execution layer.

The right exoskeleton knee-joint encoder angle is denoted uniformly by $\theta_{\mathrm{act}}$, with knee flexion defined as the negative direction in the control coordinate system. For offline analysis, the DMP reference trajectory was aligned using a fixed temporal shift. For task *m*,

(16) $$\theta_{\mathrm{DMP},m}^{\mathrm{aligned}}\left(t\right)=\theta_{\mathrm{DMP},m}\left(t-\Delta t_{m}\right),$$

and linear interpolation was used to map the shifted trajectory onto the time axis of the actual trajectory. The values of $\Delta t_{m}$ for Flat, Ramp, and Stairs were 0.181, 0.167, and 0.121 s, respectively. After alignment, $e_{i}=\theta_{\mathrm{act},i}-\theta_{\mathrm{DMP},i}^{\mathrm{aligned}}$ was calculated over all valid overlapping samples in the assisted interval, and the mean absolute error (MAE), root mean square error (RMSE), and Pearson correlation coefficient were obtained:

(17) $$\mathrm{MAE}=\frac{1}{N}\sum_{i=1}^{N}\left|e_{i}\right|,\;\mathrm{RMSE}=\sqrt{\frac{1}{N}\sum_{i=1}^{N}e_{i}^{2}}.$$

## 3. Experimental Design and Evaluation Methods

This study included an offline five-IMU locomotion-mode recognition experiment and human assistance evaluations. In the offline experiment, six subjects wore the five-IMU sensing system without the knee exoskeleton. The kinematic assistance analysis involved two subjects (S1 and S2), while the separate 10 kg load-carrying sEMG comparison involved four subjects (S1–S4). The three locomotion tasks were level walking (Flat), ramp ascent (Ramp), and stair ascent (Stairs).

### 3.1. Offline Five-IMU Locomotion-Mode Recognition Experiment

#### 3.1.1. Data Acquisition and Sample Construction

Six healthy subjects were recruited for locomotion-mode data acquisition. During this offline experiment, the subjects wore only the five-IMU sensing system and did not wear the knee exoskeleton. The five sensors comprised the Pelvis IMU and IMUs attached to the left shank, left thigh, right shank, and right thigh. The study protocol was approved by the Institutional Review Board of the Shenzhen Institutes of Advanced Technology, Chinese Academy of Sciences (SIAT-IRB-200715-H0512), and the study was conducted in accordance with the Declaration of Helsinki. All subjects provided written informed consent.

Each subject completed the three locomotion tasks. There were six source recording groups for each task, 18 source groups across the three tasks. Before data acquisition, the initial IMU orientations were calibrated while the subject stood naturally upright. For each task and subject, the continuous valid data were divided chronologically into non-overlapping training, validation, and test segments using a 6:2:2 ratio. This yielded 18 segments per task and 54 segments overall, with six segments per task in each partition. Each dataset combined the corresponding segments from all six subjects and all three tasks. Subject-independent generalization was additionally evaluated using six-fold leave-one-subject-out (LOSO) cross-validation: in each fold, one subject was held out for testing, and the remaining five subjects were used for model development. The reported LOSO values are the mean and standard deviation across the six held-out-subject folds.

Samples for the training, validation, and test sets were constructed within their respective segments using sliding windows of 200 frames with a step of 20 frames. The three datasets shared no raw frames. All models used the same original, non-augmented training windows.

Table 5 summarizes the recording duration, chronological 6:2:2 partition, and resulting windows for each terrain. Participants walked at a self-selected natural pace. Because the recognition model used fixed-length windows rather than gait-cycle-segmented samples, walking speed and gait-cycle count were not used as model inputs or evaluation units.

#### 3.1.2. Comparison Models and Evaluation Metrics

The comparison models reported in this study included linear discriminant analysis (LDA), *k*-nearest neighbors (*k*NN), a temporal convolutional network (TCN), a convolutional neural network–bidirectional gated recurrent unit (CNN-BiGRU), and a lightweight Transformer. Classification performance was evaluated using accuracy, balanced accuracy, and macro-F1.

### 3.2. Human Assistance Experiment

#### 3.2.1. Subjects and Experimental Conditions

S1 and S2, who participated in the offline data-acquisition experiment, completed the exoskeleton kinematic evaluation under unassisted and automatic-assistance conditions. Right-knee kinematics and phase-aligned DMP correspondence were evaluated without an additional external load; online DMP–knee trajectory correspondence under a 10 kg load was additionally evaluated for S1. A separate sEMG comparison under the same 10 kg load in both assistance conditions included four subjects (S1–S4). Within each paired comparison, the external-load condition was identical between the unassisted and assisted recordings. The experimental conditions are summarized in Table 6.

#### 3.2.2. Experimental Procedure

For the S1 and S2 kinematic protocol, each subject completed the three locomotion tasks under both assistance conditions, and the start and end times of each task were marked. During automatic-assistance trials, the online classifier operated continuously, and the stabilized recognized mode directly determined the DMP reference trajectory and assistance parameters applied by the control platform. For the S1 loaded-condition analysis, the online DMP reference and measured right-knee angle were recorded synchronously throughout the assisted intervals. The order of the two assistance conditions was counterbalanced across S1 and S2, with rest provided between conditions. Within each marked task period, only stable locomotion intervals containing complete gait cycles were included in the analysis. The four-subject sEMG comparison comprised paired unassisted and assisted recordings for each of the three tasks under an identical 10 kg load.

#### 3.2.3. Surface Electromyography Acquisition and Evaluation

**Figure 5:**
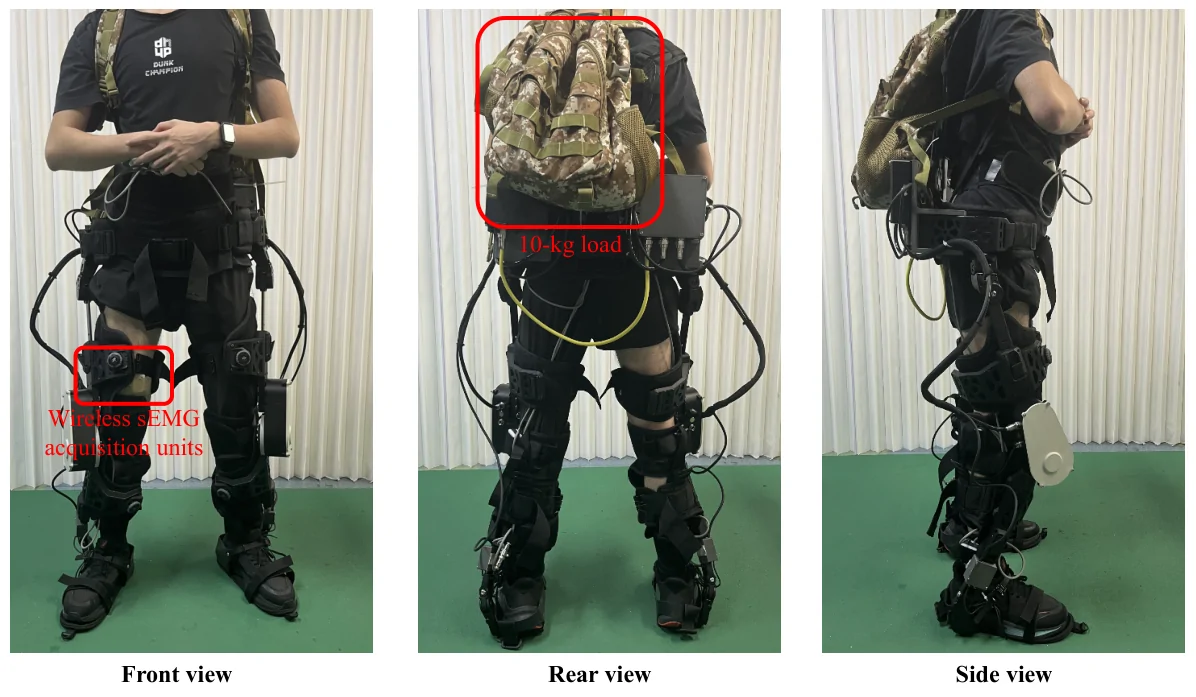
Representative wearable configuration for the 10 kg load-carrying sEMG protocol. The subject wore the knee exoskeleton, the five-IMU sensing system, and wireless sEMG acquisition units. The rear view indicates the 10 kg additional load; the front, rear, and side views are shown from left to right.

The wireless sEMG acquisition system shown in Figure 2e was used in the loaded protocol illustrated in Figure 5. Surface-electromyography signals were acquired while each subject carried an additional load of 10 kg. Signals were recorded from the right vastus lateralis (VL) and right biceps femoris (BF), corresponding to the side used for knee-trajectory analysis. VL and BF were selected as representative knee-extensor and knee-flexor muscles, respectively, so that the sEMG observations were directly related to muscle groups contributing to knee-joint control during the evaluated tasks. The electrodes were placed along the muscle-fiber direction while avoiding the exoskeleton straps, and their positions were kept unchanged between the two conditions. The electromyography sampling frequency was 2000 Hz. After mean removal, frequency components from 20 to 450 Hz were retained, while the 48–52 Hz band was excluded. The filtered signal was then reconstructed by inverse transformation. The root mean square (RMS) was calculated over all valid samples in the selected stable recording. Let the filtered electromyography samples be $s_{\mathrm{EMG}}\left(i\right)$:

(18) $${\mathrm{RMS}}_{\mathrm{EMG}}=\sqrt{\frac{1}{N}\sum_{i=1}^{N}s_{\mathrm{EMG}}^{2}\left(i\right)}.$$

The relative RMS ratio and relative change under automatic assistance were defined as

(19) $$\begin{array}{cc} \mathrm{Relative}\;\mathrm{RMS}\;\mathrm{ratio} & =\frac{{\mathrm{RMS}}_{\mathrm{assist}}}{{\mathrm{RMS}}_{\mathrm{unassisted}}}\times 100\%,\\ \Delta_{\mathrm{EMG}} & =\frac{{\mathrm{RMS}}_{\mathrm{unassisted}}-{\mathrm{RMS}}_{\mathrm{assist}}}{{\mathrm{RMS}}_{\mathrm{unassisted}}}\times 100\%. \end{array}$$

Here, $\Delta_{\mathrm{EMG}}>0$ indicates a reduction in electromyography RMS under assistance, whereas $\Delta_{\mathrm{EMG}}<0$ indicates an increase. The sEMG amplitudes were not normalized to maximum voluntary contraction (MVC). Consequently, the analysis was restricted to within-subject relative RMS changes between paired conditions and did not use absolute amplitudes for between-subject physiological comparison. For each task, the mean relative change across the measured VL and BF muscles was calculated as their equally weighted mean. The mean across the three tasks was calculated as the equally weighted mean of Flat, Ramp, and Stairs.

### 3.3. Data Presentation and Descriptive Analysis

The human assistance evaluations reported cycle-normalized right-knee kinematics, phase-aligned DMP comparison, S1 online DMP tracking under the 10 kg load, and sEMG changes. Individual and four-subject mean sEMG responses are reported descriptively; no inferential tests were performed for the assistance outcomes.

## 4. Results

### 4.1. Offline Locomotion-Mode Recognition Results

**Figure 6:**
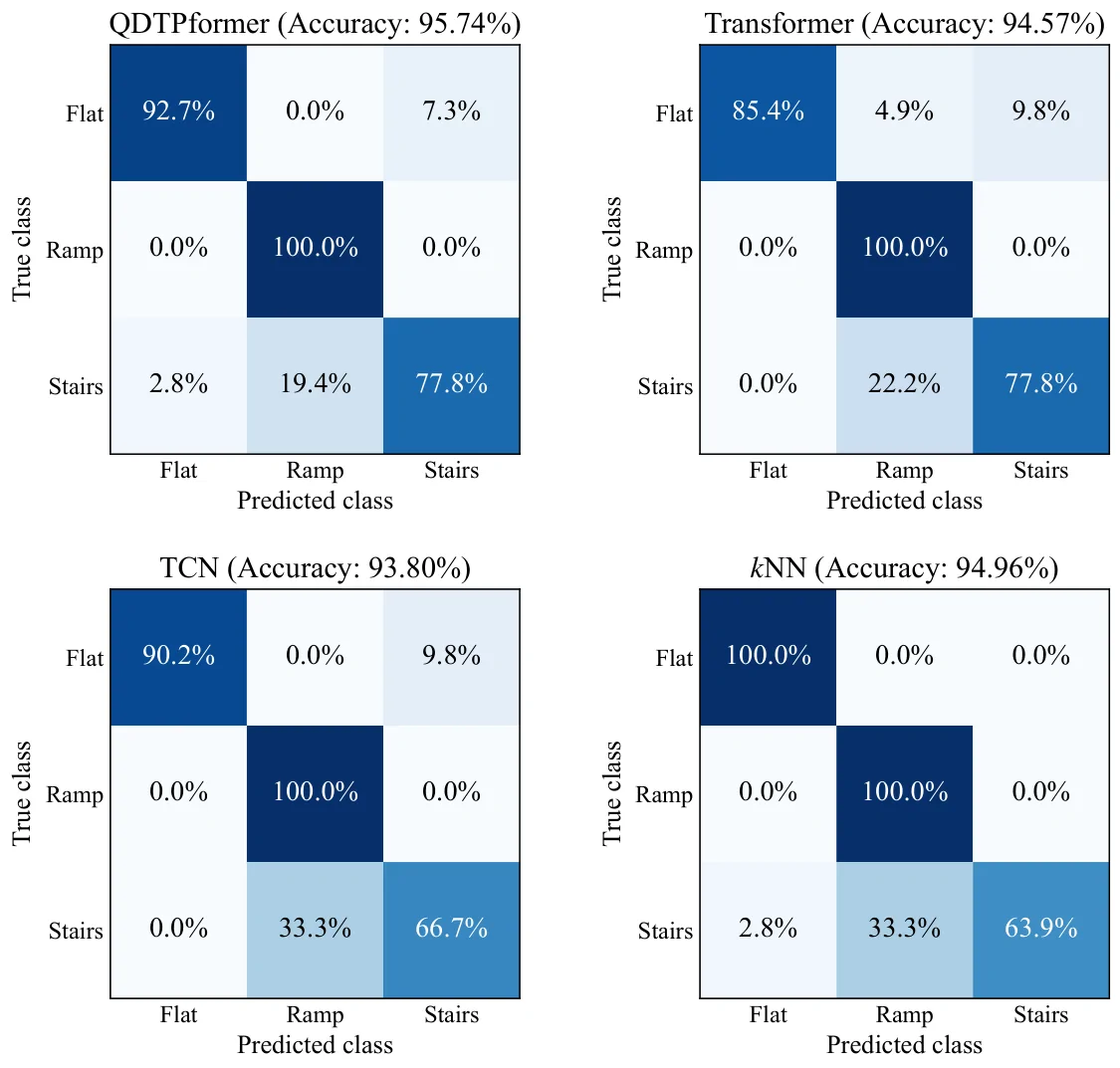
Normalized confusion matrices of QDTPformer, Transformer, temporal convolutional network (TCN), and *k*-nearest neighbors (*k*NN) on the test set. The horizontal axis denotes the predicted class, and the vertical axis denotes the true class.

QDTPformer achieved an accuracy of 95.74%, a balanced accuracy of 90.15%, and a macro-F1 of 92.23% on the test set. As shown in Figure 6, its class-wise recall values were 92.7%, 100.0%, and 77.8% for Flat, Ramp, and Stairs, respectively. The principal residual error was confusion of Stairs with Ramp (19.4%); 7.3% of Flat samples were classified as Stairs, and 2.8% of Stairs samples were classified as Flat.

Table 7 summarizes the test performance of QDTPformer and the comparison models. Compared with the Transformer, QDTPformer improved accuracy, balanced accuracy, and macro-F1 by 1.17, 2.44, and 1.64 percentage points, respectively. In the additional six-subject LOSO evaluation, the mean window-level accuracy was 89.54%±1.07%, the balanced accuracy was 79.97%±16.86%, and the macro-F1 was 76.20%±12.27% across the six held-out-subject folds.

For a single 200-frame input window, the median QDTPformer forward-pass time was 1.603 ms with an NVIDIA RTX 4090 graphics processing unit (GPU) and 2.555 ms with an Intel Core i9-14900K central processing unit (CPU). These are model-inference times.

### 4.2. Human Assistance Results

During the automatic-assistance experiment, the five-IMU locomotion recognition and decision-stabilization modules operated continuously. Each stabilized recognition output was converted online into the corresponding assistance mode. The system then selected the Flat-, Ramp-, or Stairs-specific DMP reference trajectory and assistance parameters, and the low-level controller generated the associated assistance torque. This recognition-driven control pipeline was used throughout the human assistance experiments for all three tasks.

#### 4.2.1. Right-Knee Kinematics and Phase-Aligned DMP Comparison

**Figure 7:**
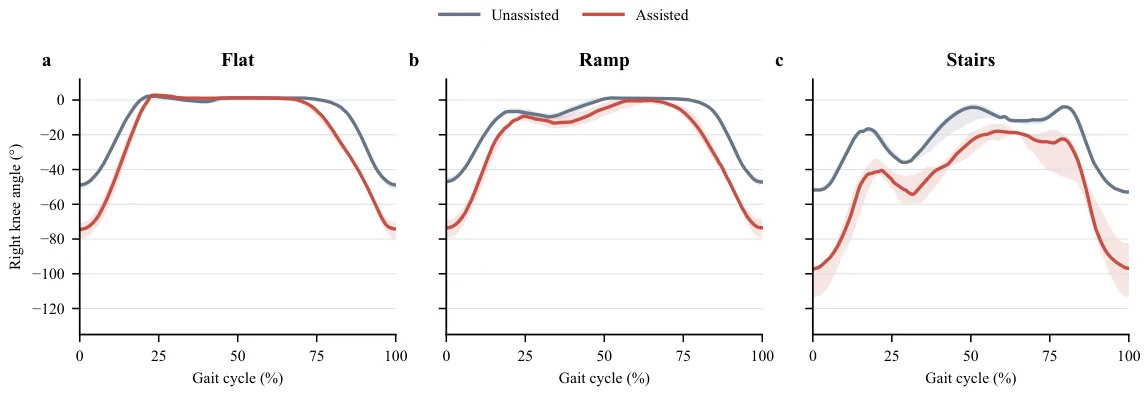
Right-knee kinematics under the unassisted and assisted conditions without an additional external load. Solid lines represent the median cycle trajectories, and shaded regions represent the interquartile ranges within each representative recording.

Figure 7 shows the median cycle-normalized right-knee trajectories and interquartile ranges from recordings in the two-subject kinematic evaluation obtained while the subjects wore the exoskeleton without an additional external load. The median peak-flexion angles for Flat, Ramp, and Stairs changed from −48.6°, −47.1°, and −52.4° in the unassisted condition to −74.2°, −73.2°, and −97.1° in the assisted condition, respectively. The corresponding within-recording changes were −25.6°, −26.1°, and −44.7°.

**Figure 8:**
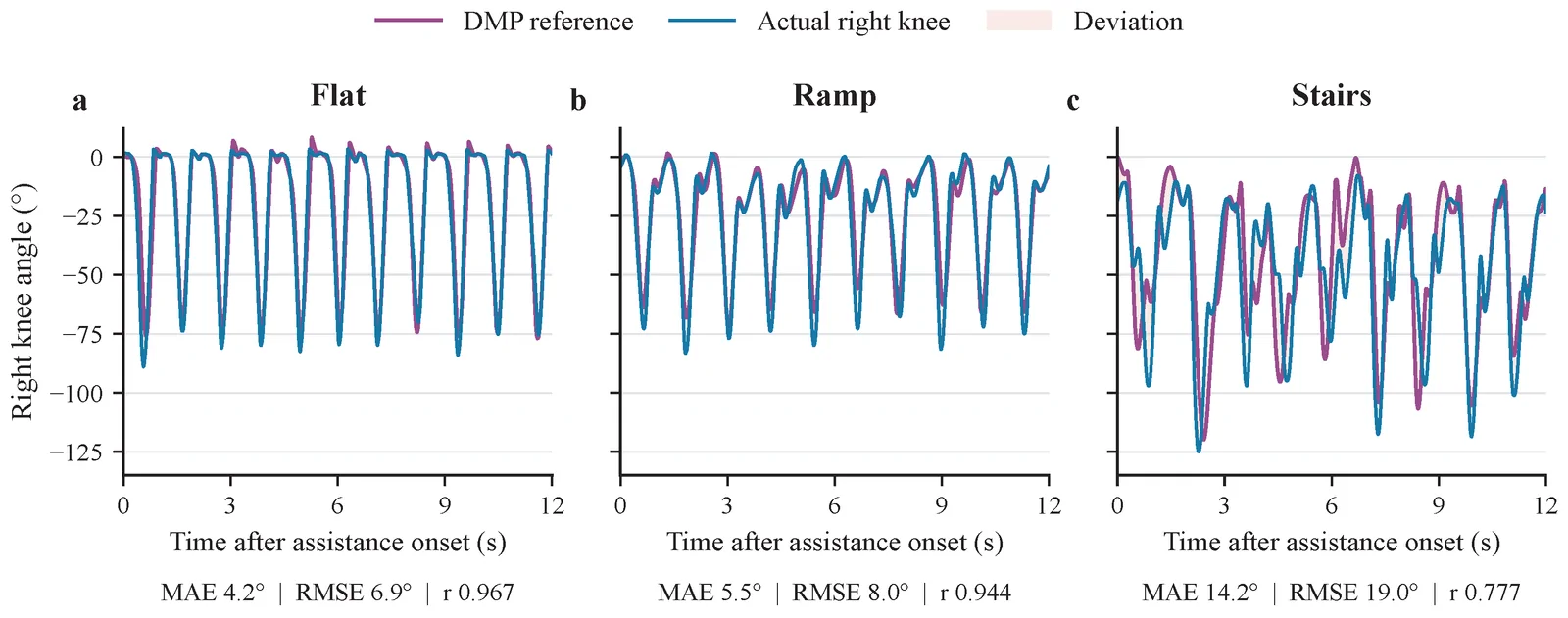
Phase-aligned DMP reference trajectories and actual right-knee trajectories from assisted recordings in the two-subject kinematic evaluation obtained without an additional external load. The lightly shaded regions indicate the instantaneous deviations between the two trajectories. DMP, dynamic movement primitive; MAE, mean absolute error; RMSE, root mean square error.

Figure 8 presents the offline phase-aligned DMP reference trajectories and actual right-knee trajectories for the three tasks. For Flat, the MAE, RMSE, and correlation coefficient were 4.2°, 6.9°, and 0.967, respectively. The corresponding values were 5.5°, 8.0°, and 0.944 for Ramp and 14.2°, 19.0°, and 0.777 for Stairs. All metrics were calculated over the complete assisted interval of the corresponding recording.

**Figure 9:**
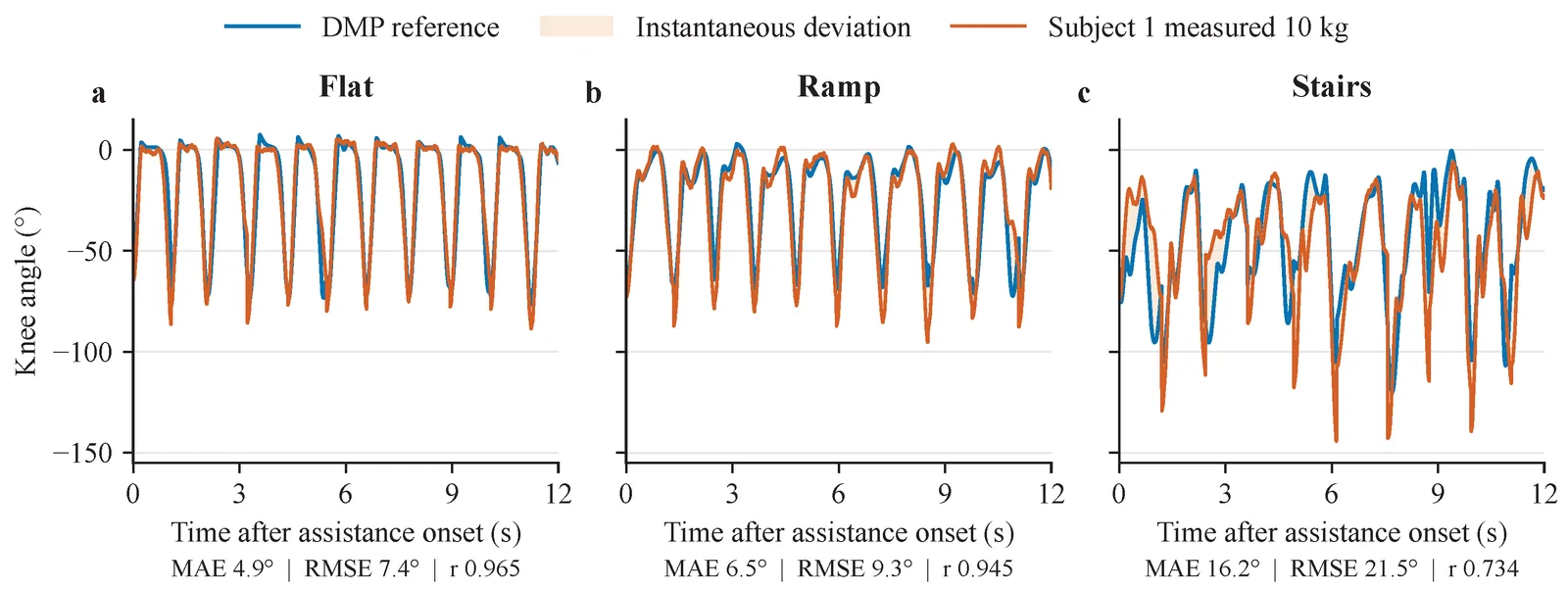
Online DMP reference trajectories and measured right-knee trajectories from S1 during assisted locomotion with an additional load of 10 kg. For visualization, representative 12 s segments are shown; the lightly shaded regions indicate instantaneous deviations between the online reference and measured trajectory. Reported metrics were calculated over the complete assisted intervals. DMP, dynamic movement primitive; MAE, mean absolute error; RMSE, root mean square error.

To assess online trajectory correspondence under external loading, Figure 9 compares the online DMP reference with the measured right-knee angle of S1 while carrying an additional load of 10 kg. For Flat, Ramp, and Stairs, the MAE values were 4.9°, 6.5°, and 16.2°; the RMSE values were 7.4°, 9.3°, and 21.5°; and the correlation coefficients were 0.965, 0.945, and 0.734, respectively. Metrics were calculated over the complete assisted interval of each recording.

#### 4.2.2. Surface Electromyography Results

**Figure 10:**
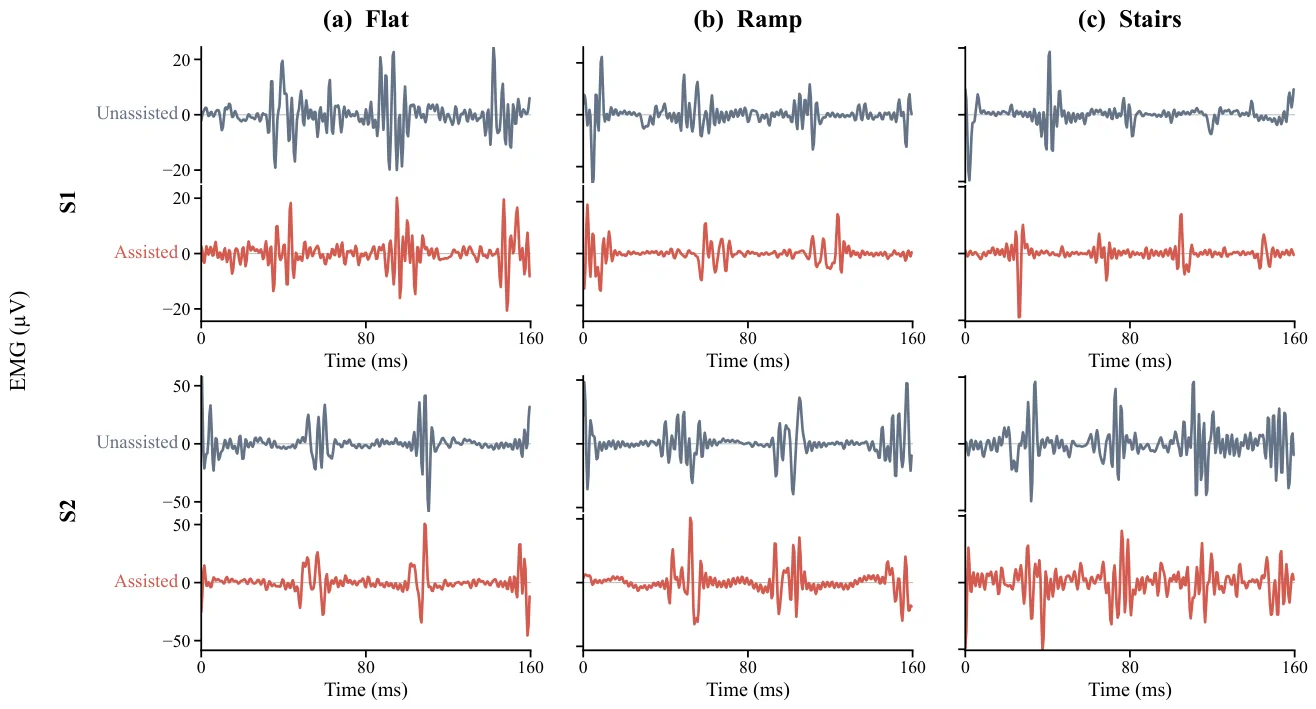
Representative 160 ms segments of the filtered right-vastus-lateralis electromyography recordings from S1 and S2 while carrying an additional load of 10 kg. In each subject–task panel, the gray upper trace and red lower trace show the unassisted and assisted conditions, respectively, using the same vertical scale.

**Figure 11:**
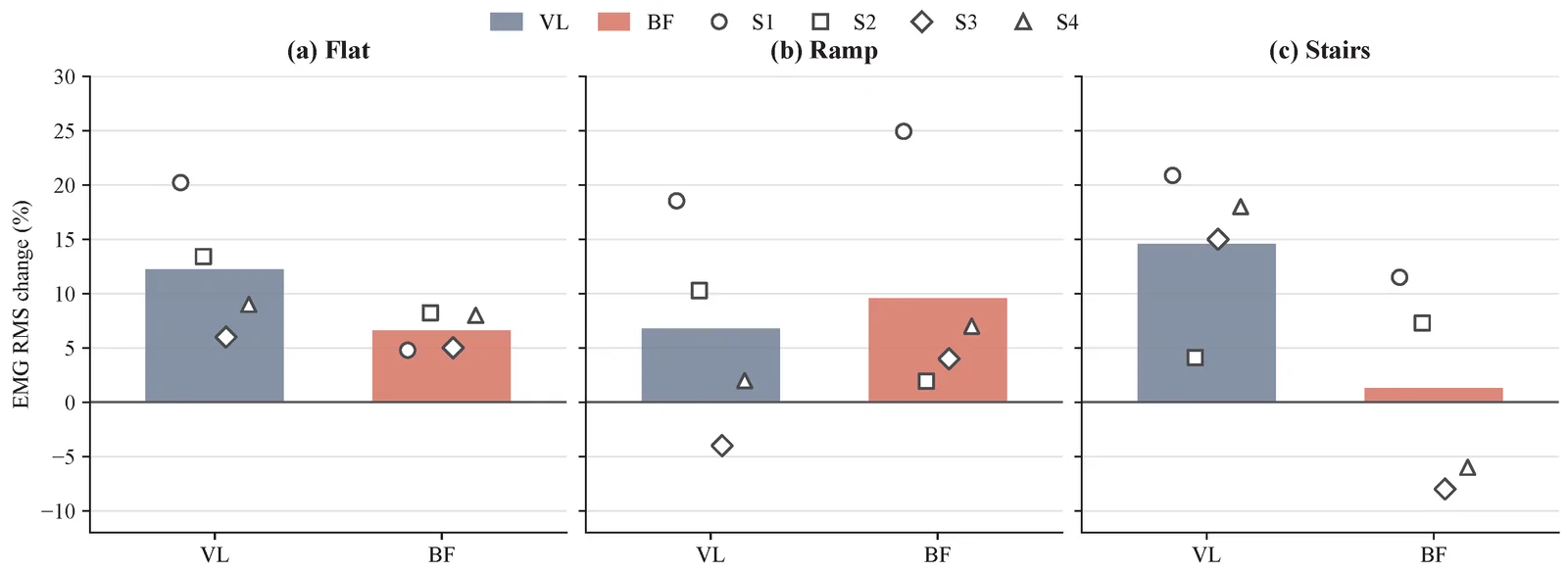
Relative changes in the root mean square (RMS) amplitudes of the right vastus lateralis (VL) and biceps femoris (BF) under assistance while carrying an additional load of 10 kg (four subjects). Blue-gray and muted-red bars indicate the four-subject means for VL and BF, respectively; circles, squares, diamonds, and triangles indicate S1, S2, S3, and S4. Positive values indicate lower electromyography RMS under assistance; negative values indicate higher RMS.

Figure 10 shows representative loaded-condition VL recordings from S1 and S2. Under the 10 kg additional load, four-subject mean relative RMS reductions for VL and BF were 12.16% and 6.51% for Flat, 6.71% and 9.47% for Ramp, and 14.50% and 1.20% for Stairs, respectively (Figure 11). The two-muscle means were 9.33%, 8.09%, and 7.85% for the three tasks; their equally weighted overall mean was 8.42%. Individual responses were not uniformly positive: S3 showed a 4.00% VL increase on Ramp, and S3 and S4 showed BF increases of 8.00% and 6.00% on Stairs.

## 5. Discussion

### 5.1. Multi-Terrain Locomotion Recognition

The normalized confusion matrix showed perfect Ramp recall but lower Flat and Stairs recall for QDTPformer; the principal residual confusion was between Stairs and Ramp. Both tasks involved pronounced lower-limb elevation, but they differed in cycle structure and intersegmental coordination. The calibrated orientation quaternions retained the overall configuration of each body segment relative to the initialized posture, whereas the adjacent-frame relative rotations emphasized local orientation changes. Their combination therefore described both the postural level and dynamic evolution within each window. QDTPformer adaptively assigned temporal weights according to the current input window and combined the resulting feature with the global average feature, thereby aggregating both locally weighted information and overall temporal information. This result is consistent with the broader use of deep temporal models in wearable locomotion recognition [18,19,20].

The normalized confusion matrices showed QDTPformer class-wise recall values of 92.7%, 100.0%, and 77.8% for Flat, Ramp, and Stairs, respectively. The largest residual confusion was Stairs classified as Ramp (19.4%), while 7.3% of Flat windows were classified as Stairs. These errors delimit the otherwise high within-cohort test-set performance, particularly for Stairs. The six-fold LOSO result (89.54% mean accuracy; 79.97% balanced accuracy; 76.20% macro-F1) is therefore reported separately as the more relevant estimate of unseen-subject performance.

### 5.2. Comparison with Recent Locomotion-Recognition Studies

Table 8 compares recent recognition studies across their sensor sets, mode sets, and validation protocols. Du et al. [26] reported 93.16% intersubject accuracy for four modes in three subjects and a mean transition delay of 1897.9 ms. Tang et al. [27] reported 97.93% mean accuracy with IMU and plantar-pressure sensing in 15 subjects and 94.44% online accuracy in five untrained subjects. Ma et al. [28] reported 99.58% accuracy for five steady modes in six subjects and a 353 ms mean transition-prediction time. The present study reports both within-cohort and LOSO performance for a five-IMU module connected to knee assistance.

### 5.3. Online Recognition and Assistance Pipeline

The automatic-assistance experiment integrated continuous five-IMU acquisition, sliding-window inference, decision stabilization, DMP reference-trajectory selection, and low-level torque control into a single online pipeline. In contrast to reporting offline classification performance alone, this pipeline further converted class probabilities into executable assistance modes. Majority voting and confidence hysteresis were used to stabilize window-level predictions, while interpolation was applied during mode changes to smooth the transition between assistance commands.

The critical integration was the direct use of the stabilized recognition result as the input for assistance-mode selection. After the recognition module estimated Flat, Ramp, or Stairs, the decision module produced a stabilized mode. This mode selected the corresponding DMP reference trajectory and assistance parameters, from which the low-level controller generated the assistance torque. The resulting chain connected online locomotion recognition to task-specific physical assistance and allowed the reference trajectory and control parameters to be adjusted separately for each task.

Median model forward-pass times were 1.603 ms on the GPU and 2.555 ms on the CPU, compared with a 0.20 s recognition-update interval; these values quantify model inference time.

### 5.4. Task-Dependent Differences in DMP–Knee Correspondence

The phase-aligned results showed closer correspondence for Flat and Ramp than for Stairs. The Stairs recording exhibited larger amplitude differences and greater local waveform variation relative to the fixed reference. Because a fixed temporal shift modifies only global timing, the remaining amplitude and shape differences were not removed by alignment.

The stable dynamical-system formulation of DMPs is suitable for generating continuous reference motions [29,30], and its temporal-scaling, amplitude, and phase parameters provide a unified form for task-specific trajectory adjustment. The phase-aligned unloaded results showed closer DMP–knee correspondence for Flat and Ramp than for Stairs (Stairs: MAE 14.2°, RMSE 19.0°). The S1 online 10 kg measurements showed a similar task pattern, with Stairs MAE 16.2°, RMSE 21.5°, and r=0.734. No adverse events or loss of balance were observed during the reported experiments. The larger stair-tracking error indicates that further trajectory adaptation is needed.

### 5.5. Muscular Responses Under a 10 kg Additional Load

Under the 10 kg load, mean VL and BF RMS decreased across the three tasks, but responses varied by participant and muscle. Ramp VL increased in S3, Stairs BF increased in S3 and S4, and the mean Stairs BF reduction was only 1.20%. These paired, within-participant changes show that assistance response was task- and wearer-dependent. Because the signals were not MVC-normalized, the analysis was restricted to paired within-participant relative RMS changes; absolute amplitudes were not compared across participants.

Human-in-the-loop and personalized systems have shown that assistance outcomes depend on wearer- and task-specific tuning [7,8,10]. Zhang et al. [12] addressed gait-control robustness, and Tricomi et al. [13] adapted hip-exosuit assistance between level walking and stairs. Together with the present sEMG variation, these studies motivate task- and wearer-specific parameter adjustment.

### 5.6. System-Level Scope, Practical Implications, and Sensor Choice

The system connects five-IMU recognition to knee assistance through decision stabilization and task-specific DMP selection. This integration is consistent with the broader emphasis on coordinated sensing and control in lower-limb robotic exoskeletons [33]. LOSO testing examined recognition in unseen participants, while the assistance experiments showed how the selected modes were executed and how knee motion and muscle activity responded.

At the high level, the terrain classifier relied on the five-IMU representation, while joint angle and torque signals were reserved for the low-level feedback/control loop. This architecture keeps the recognition representation tied to wearer segment motion and decouples the high-level terrain decision from the applied assistance state. Plantar-pressure or joint-feedback fusion could provide complementary information, particularly around transitions, but would constitute a different multimodal design and is an appropriate direction for future work.

The intended application is load-carrying assistance across terrains that require different knee trajectories. Further evaluation should include more participants and sessions, terrain transitions, and a wider range of loads and walking speeds.

### 5.7. Limitations and Future Work

The current feasibility evaluation is limited by the differing cohort sizes across assistance experiments and by the lack of cross-session and transition-specific validation. The assistance evaluations used two participants for unloaded kinematics, S1 for online 10 kg tracking, and four participants for paired 10 kg sEMG. The sEMG results are reported as paired within-participant relative RMS changes, and LOSO evaluation measures recognition in unseen participants. Future evaluation will extend the repeated-measures cohort, cross-session testing, and transition-specific timing and error analysis.

## 6. Conclusions

This feasibility study connected five-IMU QDTPformer recognition to task-specific DMP references and knee-exoskeleton assistance. QDTPformer achieved 95.74% accuracy on the within-cohort offline test set and 89.54%±1.07% mean window-level accuracy in six-fold LOSO evaluation. Online recognition selected the assistance mode in the two-subject kinematic experiment. DMP–knee correspondence was closer for Flat and Ramp than for Stairs, including in S1’s online 10 kg tracking record. In the separate four-subject 10 kg sEMG comparison, the equally weighted mean relative RMS reduction was 8.42%, with task- and wearer-dependent variation. Together, these results demonstrate the feasibility of connecting locomotion recognition to task-specific knee assistance.

## Figures and Tables

**Table 1:** Principal specifications of the quasi-direct-drive knee exoskeleton.

Parameter	Value
Exoskeleton system mass	10 kg
Knee-joint range of motion	0°–135°
Maximum joint speed	23 rad/s
Rated output torque	20 N·m
Peak output torque	75 N·m
Battery voltage	24 V
Maximum operating time	6 h
Low-level control frequency	1 kHz

**Table 2:** Placement and principal represented information of the five inertial measurement units (IMUs).

ID	Placement	Principal Represented Information
IMU 1	Posterior pelvis	Pelvic posture and overall motion trend
IMU 2	Left shank	Left-shank and knee motion
IMU 3	Left thigh	Left-thigh swing
IMU 4	Right shank	Right-shank and knee motion
IMU 5	Right thigh	Right-thigh swing

**Table 3:** Composition of the 35-dimensional IMU input feature vector for each frame.

Feature Group	Channels per IMU	Total Dimension
Calibrated orientation quaternion	$\left[q_{w},q_{x},q_{y},q_{z}\right]$	5×4=20
Adjacent-frame relative rotation	$\left[\Delta r_{x},\Delta r_{y},\Delta r_{z}\right]$	5×3=15
Total	—	35

**Table 4:** Principal configuration of the locomotion-mode recognition network.

Parameter	Setting
Input size	35×200
Embedding dimension	64
Number of Transformer encoder layers	2
Number of attention heads	4
Feed-forward dimension	128
Scoring-network dimensions	64→32→1
Encoder/classification-head dropout	0.2/0.3
Number of output classes	3
Output classes	Flat, Ramp, Stairs

**Table 5:** Frame counts, aggregate durations, and window counts for the chronological training, validation, and test partitions. Values separated by slashes are reported as training/validation/test.

Terrain	Total Frames	Duration (s)	Partition Frames	Partition Windows
Flat	17,308	173.08	10,385/3462/3461	510/164/164
Ramp	20,423	204.23	12,254/4085/4084	603/195/195
Stairs	18,205	182.05	10,923/3641/3641	537/173/173
Total	55,936	559.36	33,562/11,188/11,186	1650/532/532

**Table 6:** Assistance conditions used in the unloaded kinematic protocol and the 10 kg load-carrying sEMG protocol.

Condition	Locomotion Recognition	Exoskeleton State
Unassisted	Online operation with recognition logging	Transparent mode with no assistance torque
Automatic assistance	Online recognition of the current locomotion mode	Automatic output of the corresponding assistance torque

**Table 7:** Test-set performance of QDTPformer and the comparison models. All models used the same partition of the final six-subject dataset and the same original, non-augmented training windows. TCN, temporal convolutional network; CNN-BiGRU, convolutional neural network–bidirectional gated recurrent unit; *k*NN, *k*-nearest neighbors; LDA, linear discriminant analysis.

Model	Accuracy (%)	Balanced Accuracy (%)	Macro-F1 (%)
QDTPformer	**95.74**	**90.15**	**92.23**
Transformer	94.57	87.71	90.59
TCN	93.80	85.64	88.89
CNN-BiGRU	94.19	86.45	88.26
*k*NN	94.96	87.96	91.18
LDA	90.70	78.68	78.61

**Table 8:** Recent locomotion-recognition studies, with task, sensor, and validation differences shown alongside reported performance. IMU, inertial measurement unit; LOSO, leave-one-subject-out.

Study	Subjects	Sensors	Modes	Reported Validation/Performance
Du et al. [26]	3	On-robot inertial sensing	4	93.16% intersubject accuracy; mean transition delay 1897.9 ms
Tang et al. [27]	15	IMU + plantar pressure	5	97.93% mean recognition accuracy; 94.44% mean online accuracy in five untrained subjects
Ma et al. [28]	6	6 IMUs	5 steady modes	99.58% average accuracy after transition decision; 353 ms average transition prediction time
Present study	6	5 IMUs	3	95.74% within-cohort test accuracy; 89.54% mean six-fold LOSO accuracy

## Data Availability

The data supporting the findings of this study are available from the corresponding author upon reasonable request.
